# An Approach to Reduce Tuning Sensitivity in the PIC-Based Optoelectronic Oscillator by Controlling the Phase Shift in Its Feedback Loop

**DOI:** 10.3390/mi16010032

**Published:** 2024-12-28

**Authors:** Vladislav Ivanov, Ivan Stepanov, Grigory Voronkov, Ruslan Kutluyarov, Elizaveta Grakhova

**Affiliations:** Research Laboratory “Sensor Systems Based on Integrated Photonics Devices”, Ufa University of Science and Technology, 32, Z. Validi St., Ufa 450076, Russia

**Keywords:** optoelectronic oscillator, photonic integrated circuit, oscillations, frequency tuning

## Abstract

Radio photonic technologies have emerged as a promising solution for addressing microwave frequency synthesis challenges in current and future communication and sensing systems. One particularly effective approach is the optoelectronic oscillator (OEO), a simple and cost-effective electro-optical system. The OEO can generate microwave signals with low phase noise and high oscillation frequencies, often outperforming traditional electrical methods. However, a notable disadvantage of the OEO compared to conventional signal generation methods is its significant frequency tuning step. This paper presents a novel approach for continuously controlling the output frequency of an optoelectronic oscillator (OEO) based on integrated photonics. This is achieved by tuning an integrated optical delay line within a feedback loop. The analytical model developed in this study calculates the OEO’s output frequency while accounting for nonlinear errors, enabling the consideration of various control schemes. Specifically, this study examines delay lines based on the Mach–Zehnder interferometer and microring resonators, which can be controlled by either the thermo-optic or electro-optic effect. To evaluate the model, we conducted numerical simulations using Ansys Lumerical software. The OEO that utilized an MRR-based electro-optical delay line demonstrated a tuning sensitivity of 174.5 MHz/V. The calculated frequency tuning sensitivity was as low as 6.98 kHz when utilizing the precision digital-to-analog converter with a minimum output voltage step of 40 μV. The proposed approach to controlling the frequency of the OEO can be implemented using discrete optical components; however, this approach restricts the minimum frequency tuning sensitivity. It provides an additional degree of freedom for frequency tuning within the OEO’s operating range, which is ultimately limited by the amplitude-frequency characteristic of the notch filter. Thus, the proposed approach opens up new opportunities for increasing the accuracy and flexibility in generating microwave signals, which can be significant for various communications and radio engineering applications.

## 1. Introduction

Microwave oscillators are crucial in sensing, telecommunications, and radar systems. A critical characteristic of these devices is their phase noise level, which indicates the spectral purity of the generated signal. Traditional (based on electronic devices) approaches to generating spectrally pure signals use frequency multiplication of low-frequency signals generated by quartz or surface acoustic wave oscillators [1]. However, the higher the frequency multiplication factor, the higher the phase noise level of its output signal. This level is calculated using the formula 20 log(N), where N represents the frequency multiplication factor [2]. In addition, most electronic microwave oscillators are produced based on gallium arsenide. They, in turn, have a relatively small frequency tuning range [3,4].

Optical microwave sources offer a promising alternative to traditional sources. Among them, various optoelectronic oscillator (OEO) schemes are notable for their simplicity and cost-effectiveness [5,6]. A typical optoelectronic oscillator (OEO) setup includes a laser source, an electro-optical modulator, a notch filter, a photodetector, a microwave amplifier, and an energy storage component [7]. In addition to its clear applications in telecommunications, OEO can also be used in sensing systems as an interrogating element through frequency interrogation, enhancing the sensor system’s quality factor and improving its sensitivity and detection limit [8,9,10]. In this context, the optical sensor is a high-quality resonant structure that functions as a notch filter. Such sensors include phase-shifting Bragg gratings [11], microring resonators [12], and microdisk resonators [13]. Research into the characteristics of OEO has led to innovative solutions in interrogation, computing, and signal processing [8,9,14,15,16,17,18,19]. To compete successfully with the top electric microwave oscillators, the OEO must maintain high stability in the output signal’s frequency and have a precise tuning sensitivity. The need for frequency stabilization arises from the OEO’s long-term instability, which is influenced by environmental disturbances when its feedback loop is open [1].

Most previously developed optoelectronic oscillators (OEOs) relied on discrete optical components, which tend to be relatively large and heavy [5]. However, many applications require microwave oscillators with smaller dimensions, lower power consumption, and a wide frequency tuning range [1]. Integrated photonics technologies meet these requirements [20] by implementing the optical components of the OEO on a photonic integrated circuit (PIC). This approach allows for integrating the optical and electrical components onto a single printed circuit board [21,22]. PIC-based OEOs [2,23,24,25,26,27,28,29] have already been developed to enhance size and weight characteristics.

Integrated photonic devices are more sensitive to changes in environmental conditions, particularly temperature, than discrete optical systems. Therefore, enhancing the accuracy and stability of OEO-based oscillators and interrogators is crucial. Fortunately, the relatively small size of these devices simplifies the task of thermal stabilization [30], which is currently a focus of active research. The recent studies [31,32,33] outline the key stages in developing a new generation of compact and versatile OEOs for commercial use.

The phase noise level in modern integrated OEOs is higher than in OEOs based on discrete optical components [22]. This difference is primarily due to the lower Q-factor of the feedback loop in integrated OEOs, which results from the smaller size of the integrated elements compared to their discrete counterparts. A possible solution for this issue is to incorporate resonant structures within the OEO circuit. The microring resonator (MRR) is a promising option as it features an ultra-high Q-factor and a significant time delay for the optical signal [34,35,36,37]. These attributes make the MRR an attractive choice as a notch filter, as it ensures a low phase noise level while maintaining a compact size for the OEO circuit. However, careful design considerations are essential to minimize electrical and thermal crosstalk, which poses a significant challenge for integrated OEO systems [24,38]. A promising approach might be implementing a double feedback loop in line with the discrete solutions presented in references [39,40,41]. This method could enhance the performance of integrated OEOs while simultaneously reducing phase noise [41], lowering the specifications for the notch filter [40], and decreasing the gain of the microwave amplifier [39].

In fundamental works on optoelectronic oscillators (OEOs) [5,42], frequency tuning has been primarily achieved by altering the resonant wavelength of the notch filter. However, the frequency tuning sensitivity of OEO systems is significantly larger than that of electrical analogs [43,44]. When the optical length of the OEO feedback loop is fixed, the tuning sensitivity is determined by the OEO’s free spectral range (FSR), which is typically in the range of tens of kilohertz when utilizing an electrical notch filter. The limited tuning capability of the electrical notch filter constrains this frequency tuning range. In contrast, traditional microwave oscillators can achieve frequency tuning steps as small as Hertz fractions through phase-locked loop circuits [24,45].

An alternative method for controlling the OEO frequency, which reduces the tuning sensitivity, involves managing the phase shift (or time delay) in the OEO feedback loop using either an electric phase shifter or a tunable optical delay line. However, when implementing the OEO with discrete optical components, the delay line is typically switched, preventing smooth changes in the delay time. This approach does not allow the implementation of compact devices with a small frequency tuning step, which are necessary in modern and future wireless communication systems. To the best of our knowledge, solutions for continuous frequency tuning using optical and electrical devices have not yet been presented [24]. A promising approach is the use of an optical delay line through integrated photonics. Delay lines with the capability for continuous delay time tuning have been reported in recent studies [46,47]. Therefore, incorporating such delay lines allows for the continuous tuning of the microwave signal frequency at the OEO output.

In this paper, we introduce a novel method for continuously controlling the output frequency of an OEO by manipulating an integrated optical delay line within a feedback loop. The structure of the paper is as follows. In Section 2, we outline the analytical model for frequency control based on the parameters of the delay line. Section 3 details the basic architecture and simulation models of the delay line utilized in our study. Section 4 presents the results of the simulations. Finally, we discuss and conclude our findings in Section 5 and Section 6, respectively.

## 2. Analytical Model of the OEO’s Frequency Tuning

As described in [42], it is crucial to have a long energy accumulation time in a high-quality component of the OEO to ensure a low phase noise level. Generally, the quality factor is the ratio of the energy stored in the element to the energy lost during one cycle at the resonant frequency, multiplied by 2π [48]. The following formula determines the quality factor of the OEO loop optical part [49]:

*Q* = 2π*f*_0_*τ*, (1)

where *f*_0_ and *τ* are the natural oscillation frequency of the OEO and the time delay of the signal in the OEO optical part (or energy accumulation time), respectively. The greater the time delay *τ* of the optical part of the feedback loop, the lower the phase noise of the microwave signal as a whole [7].

The natural frequencies of the OEO are a grid of frequencies generated simultaneously in the absence of an optical notch filter, also called a frequency comb [50]. The OEO’s natural frequencies are simultaneously generated without an optical notch filter, providing a so-called frequency comb. The FSR of this comb [51] is determined by the value of the feedback loop’s time delay and is a multiple of the phase shift of 2π. So, the principle behind controlling the OEO’s output frequency lies in adjusting the phase shift Δφ [52] within the feedback loop (both in the optical and electrical domains) by altering the parameters of the delay line:

(2) $$\Delta \varphi =2\pi f_{0}\Delta \tau ,$$

and the frequency shift Δf is determined as follows [53]:

(3) $$\Delta f=-\frac{1}{2\pi \tau_{D}}\Delta \varphi ,$$

where *τ_D_* reflects the total time delay of the OEO circuit, considering the optical and electrical components.

The value of the OEO’s natural frequency *f*_0_ is determined as follows [42]:

*f*_0_ = *k*/*τ* = *kc*/*L*, (4)

where *k* is the natural number, *τ* is the time delay of the OEO optical part, *c* is the velocity of light, and *L* is the optical length of an OEO loop.

Based on these formulas, frequency shift Δ*f* caused by variation in the loop optical length Δ*L* is determined as follows:

(5) $$\frac{\Delta f}{f_{0}}=-\frac{\Delta L}{L}.$$

**Figure 1:**
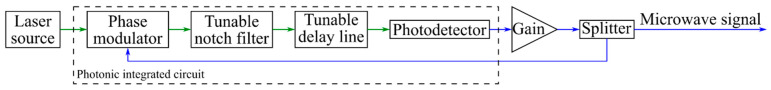
The circuit diagram of an integrated OEO supplemented with a delay line. Optical signals are displayed in green, while electrical signals are represented in blue.

Expressions (3) and (4) clearly show that increasing the optical length of the feedback loop decreases the OEO’s natural oscillation frequency. Based on the equations above, we can make the following assumption: using a continuously tunable optical delay line will allow continuous tuning of the OEO frequency within the notch filter’s bandwidth. Figure 1 shows the circuit diagram of an integrated OEO supplemented with a delay line.

A narrow-band notch filter isolates one carrier frequency (OEO output frequency), which is determined as follows [1]:

(6) $$f_{RF0}=\frac{k-\frac{\varphi_{0}}{2\pi }}{\tau_{D}},$$

where *φ*_0_ is the signal’s phase at the OEO’s output.

At the same time, the OEO output frequency, without considering the phase shift, is determined by the difference between the wavelength of the laser λ*_l_* (optical carrier) and the resonant wavelength of the notch filter λ*_notch_* as:

(7) $$f_{RF0}=f_{l}-f_{notch}=c\left(\frac{\lambda_{notch}-\lambda_{l}}{\lambda_{l}\lambda_{notch}}\right),$$

where *f_l_* = *c*/*λ_l_*, *f_notch_* = *c*/*λ_notch_*.

As mentioned earlier, a microring resonator (MRR) is frequently utilized as a notch filter in integrated photonics. Typically, adjusting the MRR’s resonant wavelength involves relatively large steps, in the order of hundreds of megahertz [54]. A tunable optical delay line for such schemas can minimize the OEO output frequency step.

The circuit in Figure 1 shows that the OEO can be considered a system with positive feedback. From a practical point of view, it is convenient to use the linear Yao–Maleki model [55] for tuning the OEO frequency by changing the delay time. In this case, the stable output signal of the OEO with an optical delay line can be calculated as a set of all circulating electromagnetic fields:

(8) $$\tilde{V}(\omega ,t)=\frac{G_{A}{\tilde{V}}_{\mathrm{in}}\exp(i\omega t)}{1-\tilde{F}(\omega )G_{A}{\tilde{V}}_{\mathrm{in}}\exp(i\omega t)},$$

where *ω* is the cyclic frequency, $\tilde{F}(\omega )$ determines the dimensionless complex transfer function of all frequency-selective elements of the circuit, *G_A_ is* the microwave amplifier gain, and ${\tilde{V}}_{\mathrm{in}}$ is the modulating electrical signal.

In the quasi-linear Yao–Maleki model [55], $\tilde{F}(\omega )$ is introduced to account for all frequency-dependent components within the OEO circuit. Therefore, based on this model, the delay line can be treated as linear. Then, the total time delay *τ_D_* of the OEO circuit at frequency *ω*, which includes the time delay for propagation along the waveguide *T*′, the tunable time delay Δ*τ*, and the group delay $\frac{d\varphi (\omega )}{d\omega }$ resulting from the dispersion of light within the waveguide, is equal to:

(9) $$\tau_{D}=T^{\prime }+\Delta \tau +\frac{d\varphi (\omega )}{d\omega },$$

where *φ* is the signal phase at the OEO output.

The group delay $\frac{d\varphi (\omega )}{d\omega }$ in (9) can be considered constant for different modes of operation of the notch filter (optical resonator) since the lengths of the integral optical waveguides connecting the elements of the OEO are relatively small. In this case, as for the Yao–Maleki model, the OEO natural frequencies are calculated using the expression

(10) $$\omega (T^{\prime }+\Delta \tau )+\varphi \left(\omega \right)+\varphi_{0}=2k\pi ,\;k=0,1,2,\ldots$$

where *k* is the OEO natural frequency index when the phase balance conditions are satisfied. Note that the frequency tuning varies periodically. Furthermore, in this paper, the time delay Δ*τ*, is considered to be introduced by the optical delay line.

Thus, taking into account expressions (9) and (10), as well as the assumption made in the Yao–Maleki linear model, FSR is inversely proportional to *τ_D_*:

(11) $$FSR=\frac{1}{\tau_{D}},$$

and the OEO natural frequencies can be determined as:

(12) $$f_{RF0}=k\cdot FSR$$

A small change Δ*τ* in the time delay of the optical signal in the OEO loop (which reflects a change in its optical length) leads to an FSR deviation and, consequently, a change in output frequency Δ*f_RF_*_0_ [9]:

(13) $$\Delta FSR=\frac{1}{\tau_{D}}-\frac{1}{\tau_{D}+\Delta \tau },$$

(14) $$\Delta f_{RF0}=k\left(\frac{1}{\tau_{D}}-\frac{1}{\tau_{D}+\Delta \tau }\right).$$

## 3. Potential Schemes for the Continuous Frequency Tuning of the OEO

In integrated photonics, two primary methods are used to control the phase shift of optical radiation: phase shift (PS) and true time delay (TTD) [56]. These methods operate by either changing the effective refractive index of the optical waveguide—often through the thermo-optical or electro-optical effects—or by discretely changing the light’s path. In the latter approach, the resulting time delay changes primarily in distinct steps. Nonetheless, some solutions allow continuous variation in the introduced time delay [46,47,57].

The primary disadvantage of the phase shift method is its dependence on the wavelength and higher losses compared to the real-time delay method [58]. Therefore, our research focuses on implementing real-time delay methods.

An integrated optical delay line can be implemented using several technologies, including a Mach–Zehnder interferometer (MZI) [59,60,61], a microring resonator (MRR) [58,62,63], a Bragg grating [47,64,65,66], a massive waveguide grating [67], and a photonic crystal [68,69]. Continuous tuning of the time delay has been demonstrated with MZIs [46,60], MRRs [70], and waveguide Bragg gratings [47]. From a production technology perspective, MZIs and MRRs are the most advantageous options [71]. Therefore, the operating principles of these elements will be discussed below.

### 3.1. MZI-Based Delay Line

**Figure 2:**
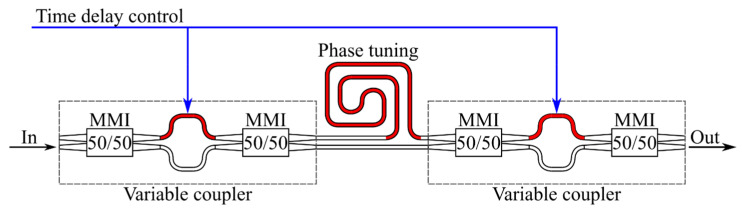
A potential implementation of an integrated optical delay line based on the MZI. The waveguides, where the effective refractive index varies, are indicated in red, while the control signal is depicted in blue.

In its simplest form, an integrated optical delay line based on an MZI consists of an unbalanced MZI comprising two tunable couplers connected by waveguides of varying lengths [46]. Figure 2 illustrates a possible implementation of this type of delay line.

The proposed implementation utilizes balanced MZIs with one controlled arm as tunable couplers. For this circuit, if the same signal is used to control the tunable couplers, the time delay τ will be determined as [46]:

(15) $$\tau =\frac{K\left(K-\cos\Delta \varphi +K\cos\Delta \varphi \right)}{2K^{2}\cos\Delta \varphi -2K\cos\Delta \varphi -2K+2K^{2}+1}T+\tau_{0},$$

where *K* represents the coupling ratio of the variable coupler; *τ*_0_ is the minimum time delay of the signal as it travels through the MZI; *T* refers to the time imbalance between the two arms of the interferometer; and Δ*φ* denotes the phase difference between the signals that pass through the different arms of the MZI.

The time delays of radiation through the longest and shortest arms of the interferometer are determined as follows:

(16) $$T=\frac{n_{g}\Delta L}{c}=\frac{1}{f_{FSR}},$$

where *n_g_* is the group refractive index of the waveguide; Δ*L* represents the difference in lengths between the arms of the unbalanced MZI; *c* is the speed of light in vacuum; and *f_FSR_* indicates the frequency difference between adjacent minima in the amplitude-frequency characteristic of the delay line.

The phase difference of signals traveling through different arms of the interferometer is equal to:

(17) $$\Delta \varphi \left(f\right)=\frac{2\pi \Delta L}{c}+\phi ,$$

where *ϕ* represents the phase shift in the most extended arm of the MZI.

Based on expression (15), we can deduce that a gradual change in the values of Δ*φ* and *K* will result in a corresponding continuous change in the introduced time delay.

### 3.2. MRR-Based Delay Line

**Figure 3:**
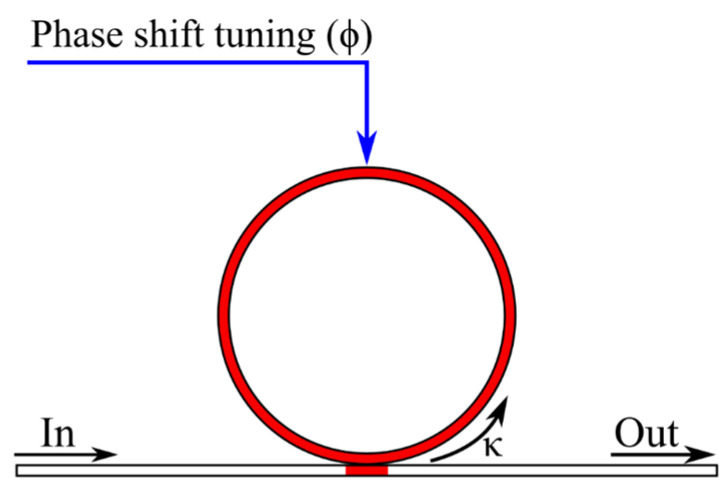
Microring resonator. Tuning elements are shown in red, and control signals are shown in blue.

Any microring resonator is a delay line [12,70]. In general, for a single MRR (Figure 3), the normalized group delay of an optical signal can be described as [57]:

(18) $$\frac{\tau \left(f\right)}{T_{r}}=\frac{\kappa }{2-\kappa -2\sqrt{1-\kappa }\cos\left(2\pi fT_{r}+\phi \right)},$$

where *κ* represents the transmission coefficient from the straight waveguide to the ring resonator; *f* denotes the frequency of the optical signal; *T_r_* is the time it takes for the optical signal to complete one round trip in the ring resonator; and *ϕ* signifies the phase shift introduced by the tuning element within the ring resonator.

In this situation, the value of *T_r_* is determined as follows:

(19) $$T_{r}=\frac{Ln_{g}}{c}=\frac{1}{f_{FSR}},$$

where *L* is the length of the ring waveguide.

To modify the time delay, one can adjust the phase shift in the ring waveguide and the coupling coefficient between the ring and the straight waveguides.

## 4. Simulation of the OEO’s Frequency Tuning Based on the Proposed Approach

The paper explores four methods for implementing integrated optical delay lines: MZIs and MRRs tuned by heating and MZI and MRR systems where the electro-optic effect alters their characteristics.

**Figure 4:**
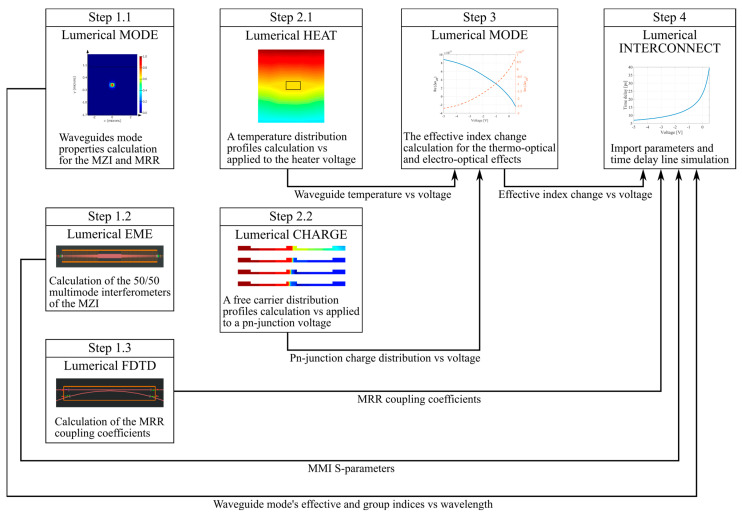
Simulation flow of time delay lines.

A generalized silicon-on-insulator platform with a waveguide layer thickness of 220 nm was employed as the integrated photonics platform. Numerical simulation was conducted using Ansys Lumerical 2020 R2 software. Figure 4 shows the numerical simulation flow of a time delay line and explains its steps.

### 4.1. Waveguide Components

**Figure 5:**
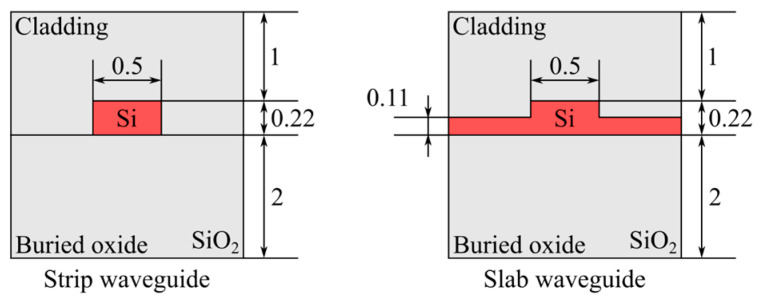
Waveguides used in numerical models (dimensions in micrometers).

We selected a waveguide width of 500 nm to ensure single-mode operation with low losses using the finite difference eigenmode (FDE) method. We used strip waveguides for thermo-optical tuning and ridge waveguides for electro-optical tuning. The geometries of the waveguides used are shown in Figure 5.

**Figure 6:**
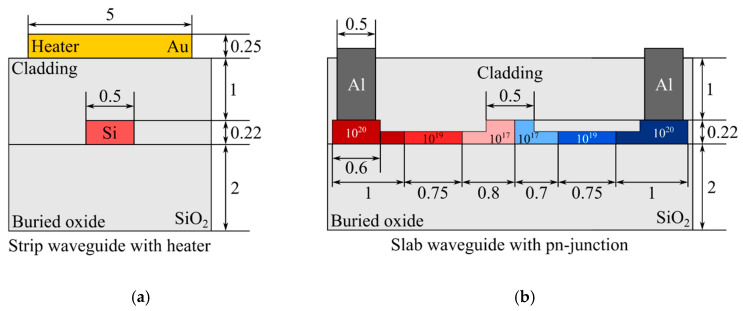
Geometric parameters of a waveguide featuring a heating element (**a**) and a semiconductor diode (**b**). The shades of red indicate regions with free carriers of the p-type, while the shades of blue represent n-type regions. All dimensions are measured in µm.

Next, we developed detailed numerical models for the heating element and the semiconductor diode integrated within the waveguide. We utilized the Ansys Lumerical DEVICE software to carry out this modeling process. Figure 6 clearly depicts the specific geometric dimensions of each component in the model, providing a visual representation of their design and configuration. The numbers in Figure 6b (placed on the waveguide cross-section) represent the carrier concentration (caused by doping), measured in m^−3^, within a waveguide with integrated pn-junction (control diode).

The primary characteristic used to describe the tuning is the change in the effective refractive index of the fundamental mode. To calculate this, we utilized two models:

- A linear model that examines how the refractive index depends on temperature for a heating element [72]:

(20) $$n+ik=\left(n_{ref}+\frac{dn}{dT}\left(T-T_{ref}\right)\right)+i\left(k_{ref}+\frac{dk}{dT}\left(T-T_{ref}\right)\right),$$

where *n* and *k* represent the real and imaginary parts of the refractive index, respectively; *n_ref_* and *k_ref_* are the real and imaginary parts of the refractive index at the reference temperature *T_ref_*, respectively, and *dn*/*dT* and *dk*/*dT* denote the change in the real and imaginary parts of the refractive index concerning temperature, respectively.

- The Soref and Bennett model at 1550 nm for a semiconductor diode [73]:

(21) $$\begin{array}{c} \Delta \alpha =\Delta \alpha_{e}+\Delta \alpha_{h}=8.88\times 10^{-21}\Delta N_{e}^{1.167}+5.84\times 10^{-20}\Delta N_{h}^{1.109},\\ -\Delta n=\Delta n_{e}+\Delta n_{h}=5.4\times 10^{-22}N_{e}^{1.011}+1.53\times 10^{-18}\Delta N_{h}^{0.838}, \end{array}$$

where Δ*α* represents the change in the radiation absorption coefficient; *e* and *h* refer to electrons and holes, respectively; Δ*N* is the change in the number of charge carriers in the active region; and Δ*n* signifies the change in the refractive index.

**Figure 7:**
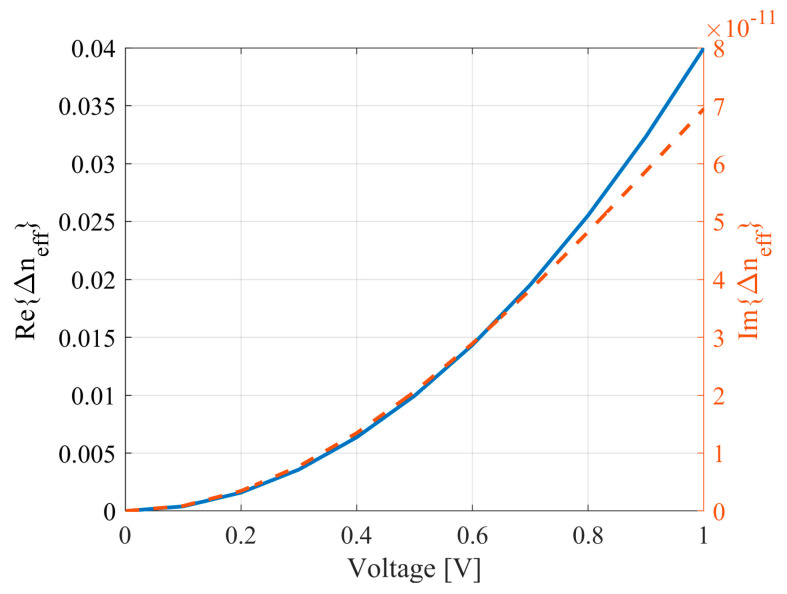
Relationship between the effective refractive index of the waveguide and the voltage applied to the heater.

**Figure 8:**
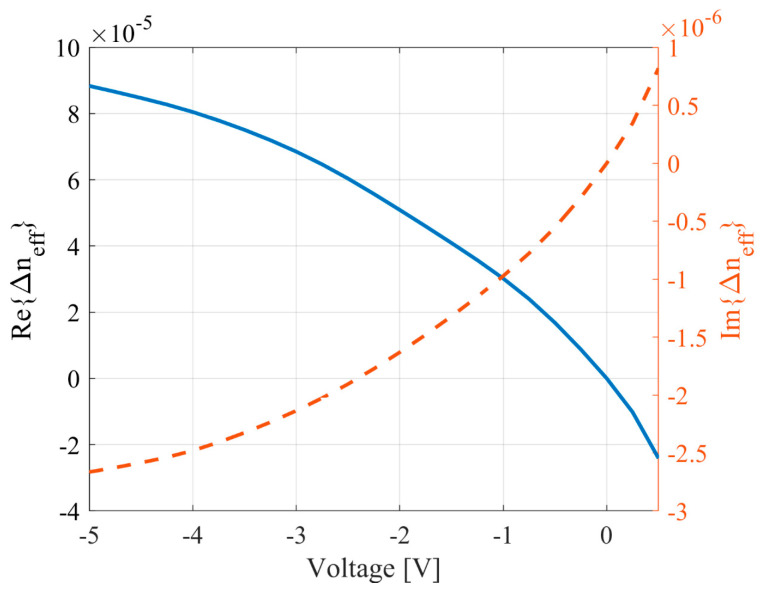
Relationship between the effective refractive index of the waveguide and the voltage applied to the anode.

Based on the relationships between temperature and charge carrier distribution in relation to voltage, as described in Equations (20) and (21), we obtained the relative dependencies of the refractive indices of waveguides on the tuning voltage. These dependencies are illustrated in Figure 7 and Figure 8. Both cases present the dependencies of the change in the refractive index relative to 0 V.

**Figure 9:**
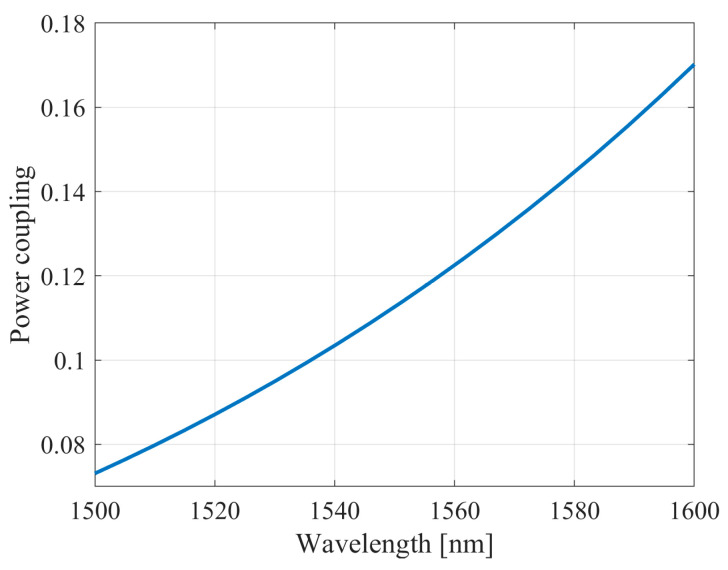
Transmission coefficient from a straight waveguide to a ring waveguide vs. the wavelength.

A multimode directional broadband coupler (MMI) operating on the self-imaging principle was simulated separately [74]. Figure 9 shows how the coupling coefficient of the coupler waveguides varies with wavelength.

### 4.2. Delay Lines

Based on the simulation results presented in the previous section, numerical models of integrated optical delay lines were developed using the Lumerical INTERCONNECT 2020 R2 software package. The following parameters were configured for the simulation:

- Gaussian pulse repetition rate—100 MHz
- simulation time—10 ns.

The sample rate significantly affects the results of numerical simulation; hence, the simulation was conducted for two values: 1.8 THz and 50 THz.

In an MZI-based electro-optical delay line, the lengths of the waveguides for the tunable couplers are set to 8 mm. The unbalanced MZI features a difference of 1 mm in the lengths of the waveguides, with the shortest waveguide measuring 0.5 mm.

In contrast, for a thermo-optical MZI, these measurements are defined as 77.2 μm, 2.5 mm, and 100 μm, respectively. Additionally, for the MRR-based delay line, the radius of the ring waveguide is 100 μm, with a gap of 180 nm. These parameters are consistent for both the thermo-optical and electro-optical MZIs.

**Figure 10:**
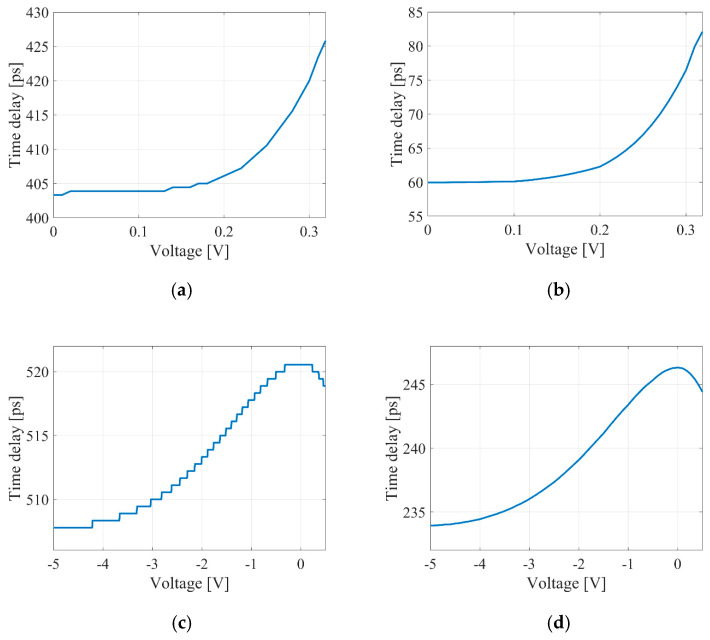
Relationship between the time delay and the applied voltage for a thermo-optical MZI at sampling frequencies of 1.8 THz (**a**) and 50 THz (**b**) and for an electro-optical MZI at frequencies of 1.8 THz (**c**) and 50 THz (**d**).

A single Gaussian pulse, limited by the simulation time window, was utilized to determine the delay time. The delay time was assessed by measuring the shift in the pulse’s maximum over time. Figure 10 illustrates the relationship between the delay time and the voltage applied to the tuning contacts.

The selected sampling frequency impacted the simulation results and increased the steepness of the graphs in Figure 10.

In addition, the model’s sampling frequency also influences the absolute value of the time delay. In the context of numerical simulation, it was found that when the sampling frequency exceeded 100 THz, the absolute value of the introduced time delay remained virtually unchanged.

Thus, the system modeling for relatively low sampling frequencies utilized an equivalent replacement of the optical delay line with an idealized component. The values for the introduced time delays were determined using the analytical expressions (15) and (18).

**Figure 11:**
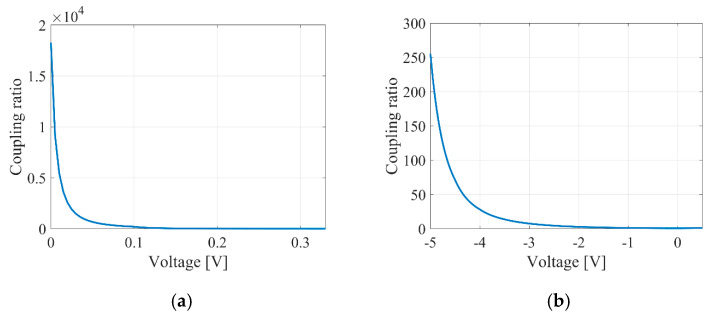
Coupling ratio of tunable couplers against the voltage applied to the tuning contact for thermo-optical (**a**) and electro-optical (**b**) MZIs.

**Figure 12:**
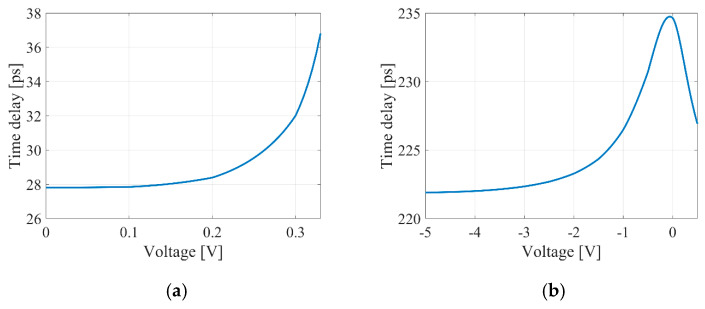
Introduced time delays against the voltage applied to the tuning contact for thermo-optical (**a**) and electro-optical (**b**) MZIs.

According to expression (15), the relationships between the ratios of the coupling coefficients of the tunable couplers and the voltage applied to the tuning contact were established for delay lines based on the MZI (see Figure 11). Additionally, the calculated relationships between the introduced time delay and the voltage for the MZI are shown in Figure 12.

**Figure 13:**
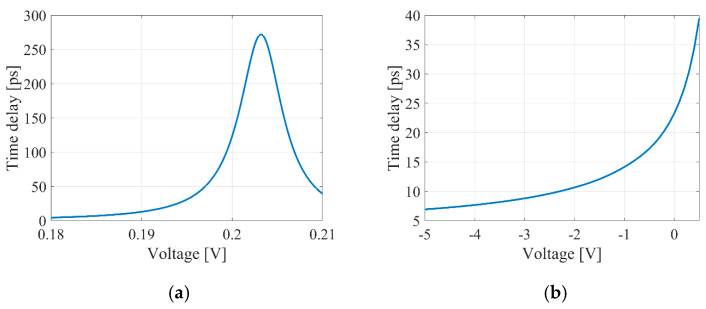
Introduced time delays against the voltage applied to the tuning contact for thermo-optical (**a**) and electro-optical (**b**) MRRs. A wavelength of 1550.4 nm was used for the calculation.

Figure 13 illustrates the calculated dependencies of the introduced time delay on voltage for the MRR.

The delay lines in the MZI offer tuning sensitivity for time delays as follows: 49.4 ps/V for the thermo-optical MZI, 8.25 ps/V for the electro-optical MZI, and 28,611.43 ps/V and 3.49 ps/V for the thermo-optical and electro-optical MRRs, respectively. These values were determined from the quasi-linear sections of the data shown in Figure 12 and Figure 13.

### 4.3. OEO Simulation

We conducted the numerical simulation according to the scheme shown in Figure 1 (with an MRR with a resonant wavelength of 1549.91 nm as a notch filter) using the following simulation parameters:

- sample rate: 1.8 THz
- simulation time window: 1 μs
- laser source central wavelength: 1550 nm for delay lines on the MZI and 1550.4 nm for delay lines based on the MRR
- laser source bandwidth: 1 MHz
- microwave amplifier gain: 85 dB

**Figure 14:**
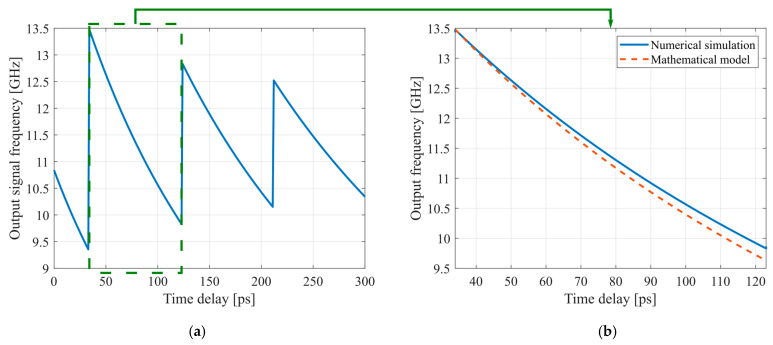
OEO’s output frequency against the introduced time delay (**a**) and comparison of the results obtained from analytical calculations and simulations (**b**). The green dashed line represents a section of the frequency curve within the 2π phase incursion.

The low sample rate results from simulation errors that occur at higher frequencies, particularly when the output signals from the photodetector exceed its cutoff frequency of 50 GHz. As mentioned previously, we replaced optical delay lines with an idealized time delay element for simulation. The value of this idealized element is independent of the sampling frequency and varies from 0 to 300 ps during the simulation. This range corresponds to a thermo-optical delay line (see Figure 13a). Figure 14a illustrates how the time delay of the optical delay line influences the OEO output frequency. Frequency value discontinuities are connected with 2π phase incursion at specific time delay values. In turn, the frequency decrease in the monotonic sections of dependence is conditioned by the total time delay value in the OEO loop. Specifically, adjusting the delay by 1 ps results in a change of 50 MHz in the OEO output frequency.

To compare the analytical model with the simulation results, we examined the range of monotonic dependence of frequency on time delay, which spans from 34 to 123 ps. Figure 14b illustrates the close agreement between the analytically and numerically obtained results.

We can determine the frequency tuning sensitivity based on the dependencies between the change in delay time, voltage, and the OEO output frequency. The thermo-optical MZI and MRR tuning sensitivity are 2470 MHz/V and 1430.5 GHz/V, respectively. In contrast, the tuning sensitivity for the electro-optical MZI and MRR are 412.5 MHz/V and 174.5 MHz/V, respectively. However, the steepness of the characteristic for thermo-optical MRR-based delay lines makes it less suitable for precise OEO frequency tuning.

## 5. Discussion

Our work aimed to demonstrate the potential of reducing the frequency tuning steps of the integrated SOI-based optoelectronic oscillator (OEO) by managing the phase shift within the processing link chain. We examined two types of integrated delay lines: those utilizing electro-optical effects and those based on thermo-optical effects. While other phase tuning mechanisms—such as interband transitions, the Pockels effect, and carrier accumulation or depletion influenced by the Franz-Keldysh effect [75]—can also be employed to control delays, they necessitate additional fabrication steps. These may include the epitaxy of different materials and modifications to the back end of the line (BEOL) [76]. Such requirements can complicate device implementation in commercial foundries.

**Figure 15:**
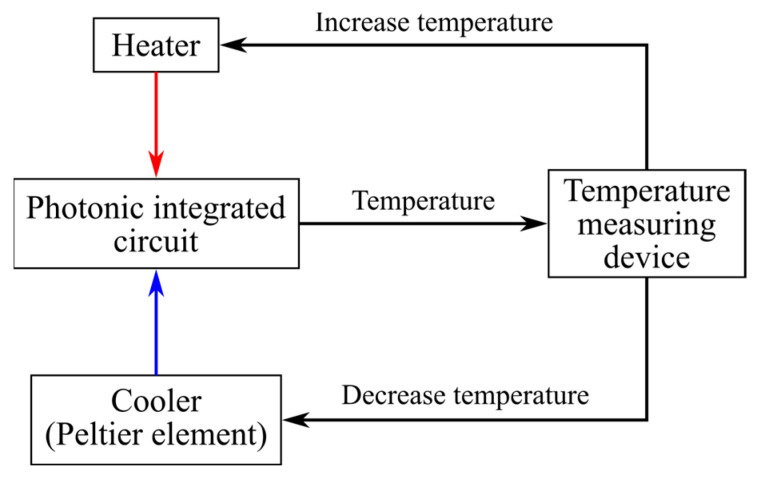
Temperature compensation scheme. The red line represents heating, and the blue one represents cooling.

The technical feasibility of the solutions we have considered needs to be addressed. As mentioned in the Introduction section, providing temperature stability presents a significant challenge when implementing solutions based on integrated photonics. Due to the high silicon temperature coefficient, as shown in [77], thermal compensation circuits are necessary. Figure 15 illustrates a block diagram of a thermal compensation mechanism that can be used for the proposed PIC. Another issue in the fabrication of PICs is the deviation in waveguide width, which can reach up to 6.4 nm [78]. However, as we noted in our previous works, waveguide width deviations of up to 8 nm can be compensated during the fabricated device’s preliminary calibration process through effective PIC temperature control [79,80]. The block diagram depicted in Figure 15 may also be applied for this purpose.

One aspect that requires additional attention during fabrication is the length of the balanced Mach–Zehnder interferometer (MZI) necessary to achieve a phase delay of 2π. In our case, an 8 mm long MZI allows for complete switching between the top and bottom waveguides. This length is significant but not impossible to realize in practice. To our knowledge, the longest electro-optic Mach–Zehnder modulator (MZM) has an arm length of 6.08 mm [81], while conventional long MZMs have arm lengths of about 4 mm [82]. Technologically, the pn-junction can be divided into segments with connected control contacts to fabricate MZIs with long arms [81,83]. Moreover, a longer active region in the MZM reduces the power required for a π phase shift [84]. It, in turn, minimizes self-heating and simplifies the implementation of the temperature compensation schemes mentioned earlier.

The final aspect of this work that may need additional discussion is using MRRs as notch filters. While other high-quality structures, such as microdisk resonators (MDRs) [85] or Bragg gratings [86], can also be employed, various research groups have focused on microring and microdisk resonators in conventional OEOs with integrated photonic devices [24]. MDRs offer a higher Q-factor, are less complex to fabricate, and are smaller than microring resonators [85]. However, they exhibit greater thermal sensitivity for MRRs of the same dimensions [85], which results in increased power consumption for temperature compensation and complicates the temperature compensation scheme.

A significant drawback of classical MDRs is resonance splitting [87], which distorts the spectral response and is undesirable for filtering applications. This issue can be mitigated by implementing a specialized MDR geometry, but such modifications require additional design considerations and complicate the fabrication process. Furthermore, the need for a vertical pn-junction in MDRs adds complexity to the manufacturing process [87]. Thus, the MRR seems to be a more suitable option for practical implementation.

In contrast, using MRRs for the time delay line is more practical since the high Q-factor of MDRs limits their operational speed [85]. We did not address this limitation in our discussion of the notch filter because the OEO frequency change period is approximately 100 ns [88], significantly exceeding the switching times of both MDRs and MRRs.

## 6. Conclusions

In this paper, we proposed a novel approach for continuously controlling the output frequency of the OEO by tuning an integrated optical delay line in a feedback loop. We introduced an analytical model, which was then verified using Ansys Lumerical software; this software precisely simulates the integrated photonic devices. The configuration of PIC components was optimized for the SOI platform. The method for continuous tuning involved two options for the true time delay lines: the Mach–Zehnder interferometer (MZI) and the microring resonator (MRR), along with two tuning principles based on either the electro-optical or thermo-optical effect.

The OEO that utilized an MRR-based electro-optical delay line demonstrated a tuning sensitivity of 174.5 MHz/V. With digital tuning control, we can estimate the minimum tuning sensitivity. Precision digital-to-analog converters (DACs) can provide a voltage step change of approximately 40 µV [89], corresponding to an output frequency step of 6.98 kHz for the OEO. We can further minimize the frequency step by improving the resolution of the DAC or employing a tuning method that uses lower voltages. The SOI platform employed in our simulation offers significant potential for the integrated development of CMOS [23] electronics, paving the way for the advancement of compact microwave oscillators.

The proposed analytical model for tuning the output frequency of the OEO can be implemented using a thermo-optical delay line [46]. It is important to note that heating the delay line does not significantly impact the notch filter when the distance exceeds 100 μm [90]. The range for temperature stabilization can be increased by modifying the architecture of the notch filter. Additionally, temperature stabilization can be achieved using circuits based on the Peltier element [64]. This approach to controlling the OEO frequency can also be implemented with discrete optical components. However, utilizing discrete components means that the optical delay line will comprise distinct segments, which limits the minimum frequency tuning sensitivity [24]. In contrast, when employing integrated photonics technologies, the delay line can function in continuous mode [91]. This capability offers greater flexibility for frequency tuning within the natural frequencies of the OEO, which are constrained by the amplitude-frequency characteristics of the notch filter.

While the proposed linear analytical model can be used to calculate the OEO output frequency considering nonlinear errors, developing a new model that accounts for the nonlinear effects typical of integrated photonics will require further development and research.

## Data Availability

The data are contained within the article.
